# A randomized prospective trial comparing clinical outcomes 3 years after surgery by Marcy repair and Prolene Hernia System^®^ repair for adult indirect inguinal hernia

**DOI:** 10.1007/s00595-012-0384-5

**Published:** 2012-10-26

**Authors:** Motohito Nakagawa, Takeshi Nagase, Tomotaka Akatsu, Shun Imai, Naoki Fujimura, Tatsuo Asagoe, Toshio Kanai

**Affiliations:** Department of Surgery, Hiratsuka City Hospital, 1-19-1 Minamihara, Hiratsuka, Kanagawa 254-0065 Japan

**Keywords:** Prosthesis, Mesh, PHS, Tension free

## Abstract

**Purpose:**

The use of mesh in the surgical repair of adult indirect inguinal hernias is widely recommended in Western countries, but no randomized controlled trials have so far been reported in Japan. The purpose of the present randomized prospective trial was to compare a mesh method with non-mesh method for surgical repair of primary adult indirect inguinal hernias in which the diameter of the internal inguinal ring was up to 3.0 cm (I-1 or I-2 of Japanese Hernia Society Classification).

**Methods:**

Patients with a primary unilateral inguinal hernia and I-1 or I-2 surgical findings were randomized to undergo either Marcy repair or Prolene Hernia System^®^ repair. Primary endpoints were recurrence, infection, and pain, with follow-up continued for 3 years postoperatively.

**Results:**

Ninety-one of 479 patients with an inguinal hernia during the study period did not meet the exclusion criteria, and 46 were allocated to Marcy repair and 45 were allocated to Prolene Hernia System^®^ repair. No recurrence was observed in either group, and no significant differences were identified between the groups in any of the primary endpoints.

**Conclusion:**

This randomized prospective trial of I-1 and I-2 inguinal hernias suggests that Marcy repair is not inferior to PHS repair. A large-scale randomized controlled trial appears warranted to confirm whether to use mesh for Japanese adult I-1 and I-2 hernias.

## Introduction

Inguinal hernias are frequently seen in clinical practice [1, 2]. Many types of treatment are available, but surgical repair is generally the most reliable method [3]. It is difficult to decide whether one particular surgical procedure leads to the best treatment outcomes, but the 2009 guidelines of the European Hernia Society (EHS) did note the high efficacy of surgery using a mesh [4]. Inguinal hernia surgery using a mesh is currently in wide use around the world, and has been recommended because of its low recurrence rate.

Inguinal hernias can be direct or indirect, and the size and location of the hernial orifice vary among patients. However, evaluation of hernia surgery is often performed for inguinal hernias in general. No evaluation of the treatment outcomes of inguinal hernia surgery using a mesh has so far been conducted based on hernia types, even in the EHS guidelines.

Evaluations of inguinal hernia surgery in randomized controlled trials have not been reported in Japan, but a retrospective study of indirect inguinal hernias did show that outcomes appear satisfactory with a Marcy repair, a type of procedure that does not use a mesh [5]. In addition, no studies have yet confirmed whether treatment outcomes for inguinal hernias described in Western countries are applicable to Japanese clinical practice.

Therefore, this study reconsidered the significance of Marcy repair for indirect inguinal hernia, which is the most common type in adult inguinal hernia in Japan [6]. Surgery without the use of a foreign body would appear preferable to surgery using a foreign body if similar treatment outcomes can be achieved. This study hypothesized that Marcy repair would not prove inferior to surgery using a mesh in primary adult indirect inguinal hernias with the size of hernia orifice defect less than 3 cm (classified as I-1 or I-2 of Japanese Hernia Society Classification [7]). This study, therefore, conducted a randomized prospective trial to compare the clinical outcomes using Marcy repair with Prolene Hernia System^®^ (PHS) repair, with follow-up continued for 3 years postoperatively.

## Methods

### Study overview

This study was conducted in adults with inguinal hernias that were scheduled to undergo elective surgery for open hernia repair under lumbar anesthesia in the Department of Surgery at Hiratsuka City Hospital between April 2002 and March 2007. Exclusion criteria included age ≤49 or ≥80 years; urgent surgery (e.g., for incarcerated hernia); surgery for recurrent hernia; concurrent bilateral surgeries; serious comorbidities; or preoperative diagnosis of a femoral hernia or a giant inguinal hernia. The nature of the study and surgery were explained to eligible patients.

Patients who provided written informed consent to participate in the study were randomized to undergo Marcy repair or PHS repair if the findings at surgery showed a type I-1 or I-2 hernia based on the Japanese Hernia Society Classification. Allocation to Marcy repair or PHS repair was determined using a computer-generated random number table. Office personnel in the Surgery Outpatient Department at Hiratsuka City Hospital were in charge of separating envelopes in order of enrollment. The attending surgeon telephoned the Surgery Outpatient Department from the operating room during surgery in order to request that the envelope be opened, and confirmed the procedure. Any change in the procedure after allocation was made by the attending surgeon if deemed necessary on an intention-to-treat basis. The patient and hospital personnel were informed of the procedure performed postoperatively.

All study protocols were approved by the Ethics Committee at Hiratsuka City Hospital in February 2002. The study is registered with Clinical Trials Registry of University Hospital Medical Information Network (UMIN-CTR, study ID: 000000604).

### Surgery

Preanesthetic medication that included 0.2 mg buprenorphine hydrochloride and 0.5 mg atropine sulfate was given to the patient by intramuscular injection when leaving the hospital ward. Sodium sulbactam/sodium ampicillin (1.5 mg) was administered intravenously just before surgery, as prophylactic antibiotic therapy. The skin of the groin was incised 6–8 cm, and then the fascia of the external oblique muscle was incised to open the inguinal canal. The spermatic cord was then dissected, and the hernia sac was opened if a type I (indirect) hernia was identified. The size of the hernia orifice defect was measured to confirm an I-1 or I-2 hernia, and then an index finger was inserted through the internal inguinal ring to palpate the posterior wall confirming no coexisting type II (direct) hernia, and finally the patient was allocated to a repair group.

Marcy repair was performed as described by Nyhus [8]. The hernia sac was ligated after sufficient dissection. The transverse fascia was then sutured to an extent that the newly made inguinal ring was tight enough just for a single Pean forceps to pass through the ring along the spermatic cord. PHS repair was performed as described by Gilbert [9]. A sufficiently large preperitoneal cavity was dissected, the underlay patch was inserted and spread, and the connector was placed in the internal inguinal ring. The spermatic cord was passed through a slit that was made, and the overlay patch was spread to cover a sufficient area of the posterior wall of the inguinal canal. This was anchored medially with 2 sutures to the firm fascia on the anterior surface of the pubic bone. In addition, the patch was anchored to the transversalis fascia, internal oblique muscle fascia, and inguinal ligament using a total of 6 or 7 sutures.

The distal sac was opened as much as possible in both types of procedures, and every effort was made to preserve the ilioinguinal and iliohypogastric nerves. The nerves were transected only in cases where the surgical procedure was otherwise impeded. The surgeon in charge of the study was fully experienced in inguinal hernia surgery and board-certified as both a general surgeon of the Japan Surgical Society and a gastrointestinal surgeon of the Japanese Society of Gastroenterological Surgery. This surgeon took part in all surgeries for patients participating in this study as the lead surgeon or first assistant to ensure uniform surgical technique.

### Postoperative follow-up and evaluation

Analgesics were administered after surgery, upon the request of the patient, and their use was recorded. No restriction was placed on activity after the effects of lumbar anesthesia wore off. Patients were usually discharged from the hospital on postoperative day 4. Recurrence, pain, and infection were evaluated as primary endpoints after 1 week, 1 month, 6 months, and then every 6 months up to 3 years postoperatively. Hernia recurrence was defined as a palpable, reducible lump in the treated groin, with or without symptoms. The patients were asked whether they had pain at rest in the treated groin. Wound infection was diagnosed by discharge of pus from the wound. Secondary endpoints, such as postoperative complications, included evaluation of hematoma, seroma, wound swelling (obviously swollen lesion in the treated groin), and testicular symptoms at 1 day, 3 days, 7 days, 1 month, 6 months, and 1 year after surgery. The peripheral blood white blood cell count, neutrophil count, and levels of C-reactive protein (CRP) and fibrinogen were assessed preoperatively and on days 3 and 7 postoperatively as an objective evaluation of the inflammatory response to the surgery. A survey was performed using an 11-grade visual analogue scale at 1 week, 1 month, and 1 year postoperatively, as a subjective evaluation of surgery. Postoperative follow-up was performed by the surgeon in charge of the study, who knew the allocated surgical procedure. Primary endpoints and postoperative complications were assessed by history and physical examination.

### Statistical analysis

Enrolling a sufficient number of patients to prove the current hypothesis would be difficult at a single institution. However, a multicenter study design was avoided to ensure strict uniformity of the surgical procedures. The goal was to enroll about 50 patients in each group. The goal was that the results of the study could be useful for a meta-analysis as a study with strictly uniform surgical procedures. The mean annual number of inguinal hernia surgeries at this hospital suggested that an enrollment period of about 4 years would be required, but the actual enrollment period was 5 years.

The results, based on the characteristics of the numerical data, were analyzed using two-sample *t* test, Fisher’s exact test, the CMH2 test, and Wilcoxon’s test. All analyses were performed on an intention-to-treat basis using the SAS version 8.2 software package (SAS Institute, Cary, NC, USA).

## Results

### Patient flowchart (Fig. 1)

A total of 479 inguinal hernia surgeries were performed between April 2002 and March 2007. One hundred and eighty-nine of those patients were judged eligible to participate in this study and received an explanation of the study purposes and methods.

**Fig. 1:**
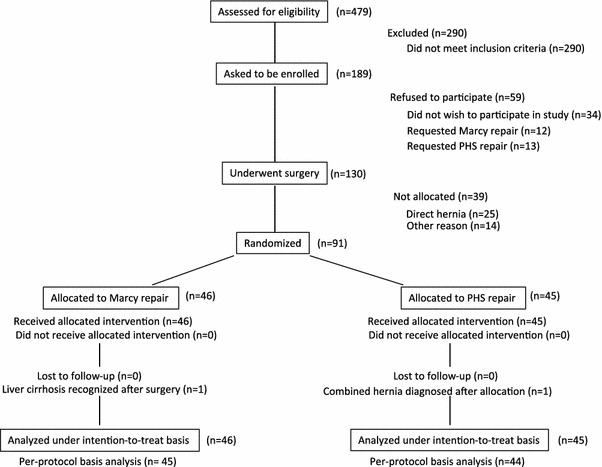
Trial profile. *n* number of patients, *PHS* Prolene Hernia System^®^

One hundred and fifteen of the 290 patients who were excluded were ≤49 or ≥80 years old, 61 required urgent surgery (e.g., for incarcerated hernia), 21 underwent surgery for recurrent hernia, 26 underwent concurrent bilateral surgeries, 57 showed serious comorbidities, 3 had a preoperative diagnosis of a femoral hernia, 6 had a giant inguinal hernia, and 1 was not Japanese.

Comorbidities included diabetes in 10 patients, chronic liver dysfunction (including cirrhosis) in 13 patients, cardiac insufficiency in 2 patients, neuropsychological disorder in 8 patients, asthma in 6 patients, cancer or cancer surgery within the previous year in 11 patients, treatment for coronary artery disease within the previous year in 5 patients, stroke within the previous year in 2 patients, postoperative prostate cancer in 8 patients, hematological disorder in 2 patients, lumbar anesthesia unsuitability in 10 patients, and other concomitant surgery in 3 patients (some patients showed overlap of these comorbidities).

One hundred and thirty of the 189 patients who received an explanation about the study expressed a desire to participate in the study. Thirty-four of the 59 patients who refused to participate did not like the idea of participating in the study, 12 patients specifically wanted to undergo Marcy repair, and 13 patients specifically wanted to undergo PHS repair.

Ninety-one of the 130 patients, who consented to study participation and underwent surgery, underwent the randomization process. Twenty-five of the 39 patients who were not randomized had a type II (direct) hernia. The remaining 14 patients included 4 patients with type I-3 (indirect hernia in which the diameter of the internal inguinal ring is larger than 3.0 cm), 3 patients with type IV (combined hernia), 3 patients with concomitant hydrocele of the spermatic cord, 2 patients with conversion to general anesthesia due to the insufficient effects of lumbar anesthesia, 1 patient with liver cirrhosis recognized after starting surgery, and 1 patient for whom surgery was started in the evening, so the personnel in charge of the study at the Surgical Outpatient Department were unavailable at the time of allocation.

Forty-six of the 91 patients who received randomization were allocated to Marcy repair and 45 were allocated to PHS repair. One patient allocated to Marcy repair was found to have liver cirrhosis on postoperative day 1. One patient allocated to PHS repair was found to also have type II (direct) hernia later during surgery. These two patients received surgery as allocated, and were analyzed as allocated on an intention-to-treat basis in the subsequent analysis. All of the allocated patients completed the 3-year follow-up to analyze the results of the primary endpoints. An analysis was also performed on a per-protocol basis, but those results yielded the same conclusions as the intention-to-treat analysis.

### Patient characteristics and surgical and perioperative outcomes (Table 1)

No differences were seen between the groups in the age, sex, body mass index, laterality of hernia, or hernia type. The operative time with PHS repair was significantly longer than with Marcy repair, but no difference was seen in the postoperative use of analgesics.

**Table 1:** Patient characteristics and operative and perioperative outcomes

	Marcy repair (*n* = 46)	PHS repair (*n* = 45)	
Age (years)	64 ± 8.1 (mean ± SD)	63 ± 7.7 (mean ± SD)	
Sex			
Male	41	41	
Female	5	4	
BMI	22.5 ± 2.6 (mean ± SD)	22.8 ± 2.5 (mean ± SD)	
Laterality			
Right	26	20	
Left	22	25	
Hernia type			
I-1	4	2	
I-2	38	35	
I-3	4	7	
IV	0	1	
Operative time (min)	68 ± 19 (mean ± SD)	84 ± 18 (mean ± SD)	*p* < 0.05
Number of times analgesics were used	0 (0–5) [Median (range)]	0 (0–2) [Median (range)]	

*PHS* Prolene Hernia System^®^, *n* number of patients, *SD* standard deviation, *BMI* body mass index

### Recurrence, pain, and infection within 3 years after surgery (Table 2)

No recurrences occurred in either group during the follow-up period. About two-thirds of the patients in both groups complained of pain at least once during the follow-up, but no differences were evident between the groups. Most complaints of pain in both groups were recorded at 1 week and/or 1 month after surgery. Three patients with Marcy repair and 2 patients with PHS repair complained of pain for ≥6 months. Only 1 patient with Marcy repair reported pain lasting >1 year. Infection only occurred in 1 patient who had undergone Marcy repair.

**Table 2:** Recurrence, pain, and infection within 3 years after surgery

	Marcy repair (*n* = 46)	PHS repair (*n* = 45)
Recurrence	0	0
Pain	30	31
Infection	1	0

Patients were included if the symptom was confirmed at least once during follow-up

*PHS* Prolene Hernia System^®^, *n* number of patients

### Complications within 1 year after surgery (Table 3)

Table 3 shows the complications judged to have occurred at least once within 1 year postoperatively. Wound swelling was more common with PHS repair than with Marcy repair. All swelling occurred within 1 week of surgery in both groups. No swelling was observed in either group ≥1 month after surgery.

**Table 3:** Incidence of complications within 1 year after surgery

	Marcy repair (*n* = 46)	PHS repair (*n* = 45)	
Hematoma	2	1	
Seroma	1	1	
Wound swelling	1	8	*p* < 0.05
Testicular symptoms	0	2	

Patients were included if the complication was confirmed at least once during follow-up

*PHS* Prolene Hernia System^®^, *n* number of patients

### Blood test data (Table 4)

Only the mean CRP level on postoperative day 3 was significantly higher in the PHS group than in the Marcy group. The CRP levels in the PHS group decreased to the same level as in the Marcy group by postoperative day 7.

**Table 4:** Blood test data

	Marcy repair (*n* = 46)	PHS repair (*n* = 45)	
WBC (/mm^3^)			
Preoperative	6537 ± 1713 (*n* = 46)	6428 ± 2112 (*n* = 45)	
PO day 3	7684 ± 1815 (*n* = 45)	7380 ± 1704 (*n* = 45)	
PO day 7	6342 ± 1601 (*n* = 45)	6267 ± 1816 (*n* = 43)	
PMN (/mm^3^)			
Preoperative	3931 ± 1303 (*n* = 45)	3698 ± 1350 (*n* = 44)	
PO day 3	4739 ± 1368 (*n* = 41)	4518 ± 1337 (*n* = 42)	
PO day 7	3658 ± 1145 (*n* = 41)	3532 ± 1107 (*n* = 40)	
CRP (mg/dl)			
Preoperative	0.1 ± 0.1 (*n* = 44)	0.1 ± 0.2 (*n* = 44)	
PO day 3	3.3 ± 2.5 (*n* = 44)	4.3 ± 2.3 (*n* = 45)	*p* < 0.05
PO day 7	0.9 ± 2.3 (*n* = 42)	1.1 ± 1.6 (*n* = 42)	
Fibrinogen (mg/dl)			
Preoperative	314 ± 73 (*n* = 45)	310 ± 57 (*n* = 45)	
PO day 3	508 ± 99 (*n* = 42)	507 ± 89 (*n* = 42)	
PO day 7	468 ± 110 (*n* = 42)	468 ± 88 (*n* = 42)	

Values represent mean ± standard deviation

*PHS* Prolene Hernia System^®^, *WBC* white blood cell, *PMN* polymorphonuclear leukocyte, *CRP* C-reactive protein, *n* number of patients, *PO* postoperative

### Survey results (Table 5)

The VAS showed that subjective symptoms of pain, numbness, difficulty walking, and overall satisfaction with treatment did not differ between groups.

**Table 5:** Survey results

	Marcy repair (*n* = 46)	PHS repair (*n* = 45)
Pain		
PO 1 week	2.8 ± 1.9	3.5 ± 1.9
PO 1 month	1.6 ± 1.8	1.6 ± 1.9
PO 1 year	0.5 ± 1.2	0.4 ± 1.1
Numbness		
PO 1 week	0.8 ± 1.5	0.8 ± 1.5
PO 1 month	0.7 ± 1.5	0.8 ± 1.5
PO 1 year	0.5 ± 1.6	0.3 ± 0.7
Difficulty walking		
PO 1 week	2.0 ± 1.9	2.2 ± 0.1
PO 1 month	0.7 ± 1.4	0.8 ± 1.4
PO 1 year	0	0.1 ± 0.5
Overall satisfaction		
PO 1 week	1.2 ± 1.6	1.6 ± 1.9
PO 1 month	0.8 ± 1.4	1.1 ± 1.6
PO 1 year	0.2 ± 0.6	0.2 ± 0.7

Values represent mean ± standard deviation of visual analogue scale scores

*n* number of patients, *PO* postoperative

## Discussion

This study demonstrated that Marcy repair may possibly provide clinical outcomes that are not inferior to PHS repair for elective surgery of primary, unilateral, adult inguinal hernias categorized as type I-1 or I-2 of Japanese Hernia Society Classification, based on follow-up for 3 years postoperatively. Tension-free procedures using a mesh are now widely used in hernia surgery. However, attention should be redirected to the existence of hernias treatable with surgery not using mesh. Surgery without the use of a foreign body would appear preferable to surgery using a foreign body, if similar treatment outcomes can be achieved [10–12].

The EHS guidelines on inguinal hernias in adult patients recommended the Lichtenstein technique as an open mesh procedure in primary unilateral hernias in July 2009 [4]. This does not negate procedures using other kinds of mesh for which only short-term results are available, but almost completely denies the significance of performing Marcy repair, which does not use a mesh. However, a detailed examination of the papers cited in the guideline that reported poor outcomes for Marcy repair reveals several potential problems [13].

Therefore, comparing Marcy repair and repair using a mesh with a strict study protocol would be meaningful. The current study compared Marcy repair with PHS repair that was the commonly performed mesh repair in this hospital at the time of planning the study protocol. In addition, this prospective trial was conducted at a single medical institution with an emphasis on strict study eligibility criteria and achieving a uniformly high level of surgical quality, even if a sufficient number of patients for performing a statistically reliable analysis could not be recruited.

No recurrences were observed during a 3-year follow-up period in either the Marcy repair or PHS repair groups. Even though Marcy repair is performed for patients with an intact posterior wall of the inguinal canal in Western countries, many recurrences have been reported on long-term postoperative follow-up [13]. This represents the basis for the negative view of Marcy repair in the EHS guidelines. However, extrapolating the findings of the report to the treatment of adult inguinal hernias in Japan may be risky with respect to the following two points. The first is a high recurrence rate of 2 % within 1 year after Marcy repair for indirect inguinal hernia with an intact posterior wall. In addition, the authors of the report estimated that half of the cases of patient death would have experienced recurrence by 15 years in calculating the recurrence rate at 15 years after surgery. Clinical outcomes in Japanese facilities aggressively performing Marcy repair are closer to the current study results [5], rather than to the results of the report. Perhaps, the EHS guideline should not be accepted as applicable to Japan because they are based on clinical outcomes in Western countries. Pain and infection also did not differ between groups in the current series. No obvious differences in the three primary endpoints were seen between Marcy repair and PHS repair.

On the other hand, some parameters showed differences between the groups. Wound swelling and elevated CRP level on postoperative day 3 (measured as a marker of inflammatory response) were both significantly more common with PHS repair. The surgical invasiveness to the body is greater in PHS repair than in Marcy repair, given the wider area of dissection and longer operative time. However, all the above parameters in PHS repair returned to the same level as in Marcy repair by 1 week after surgery. Furthermore, the postoperative use of analgesics and patient survey results did not differ between the groups. The results of these secondary endpoints support the fact that Marcy repair is at least not inferior to PHS repair.

Some weaknesses in the design of the present study must be considered. They are the blindness of the procedure and the duration of follow-up [14]. A double-blinded protocol and follow-up period exceeding 3 years could have resulted in the failure to complete the trial in a single hospital setting. Therefore, the repair method was revealed and the follow-up was finished at 3 years after surgery. Further follow-up should be performed to confirm the long-term results. Another weak point in the design was the small number of patients due to the strict selection criteria of patients, which made statistically reliable results difficult to obtain. Some investigators may argue that the patient group is too highly selected and represents only a minority of all inguinal hernia patients who have various backgrounds. However, the ideal sample to compare the outcomes of two methods of surgery is two identical groups. Therefore, the current study applied strict selection criteria to utilize elective surgeries of adult primary unilateral indirect inguinal hernia patients who had no serious comorbidities. The objective was to obtain results that would be useful for future planning of a large-scale high-quality clinical trial.

General surgeons often deal with the treatment of various solid cancers for which postoperative recurrence means that radical cure cannot be achieved. However, the postoperative recurrence of inguinal hernia is treatable. No recurrence is of course ideal, but surgery using a mesh may not be universally the best strategy for further reduction of the low recurrence rate if a very small number of recurrences with a surgery not using a mesh is acceptable. Some patients prefer surgery not using a foreign body, thus suggesting the idea for this study. The current results certainly support this idea.

In conclusion, although the number of enrolled patients was too small due to the establishment of strict eligibility criteria and a uniformly high level of surgery, the results of this randomized prospective trial may suggest that the treatment outcomes with Marcy repair are not inferior to those with PHS repair in adult patients with primary unilateral indirect inguinal hernias with a follow-up for 3 years postoperatively. Investigation of whether major differences arise with use or nonuse of a foreign body is meaningful for patients with adult indirect inguinal hernias. A large-scale, high-quality clinical trial should thus be conducted in the future.

## References

[1] Rutkow IM. Epidemiologic, economic, and sociologic aspects of hernia surgery in the United States in the 1990s. Surg Clin North Am 1998;78:941–51; v–vi. 10.1016/S0039-6109(05)70363-7 9927978

[2] Primatesta P, Goldacre MJ (1996). Inguinal hernia repair: incidence of elective and emergency surgery, readmission and mortality. Int J Epidemiol.

[3] Chunq L, Norrie J, O’Dwyer PJ (2011). Long-term follow-up of patients with a painless inguinal hernia from a randomized clinical trial. Br J Surg.

[4] Simons MP, Aufenacker T, Bay-Nielsen M, Bouillot JL, Campanelli G, Conze J (2009). European hernia society guidelines on the treatment of inguinal hernia in adult patients. Hernia.

[5] Kawamura T, Sato K, Sahara H, Oishi Y, Nakajima A (2009). Clinical outcomes by Marcy repair for type I-1 and I-2 inguinal hernia based on the Japanese Hernia Society Classification (in Japanese). Nihon Gekakeirengo Gakkaishi (J Jpn Coll Surg).

[6] Sakurai S (2009). Groin hernia classification and related anatomy (in Japanese). Shokakigeka (Gastroenterol Surg).

[7] Japanese hernia society. Japanese hernia society classification of inguinal hernia. 2009. http://www.med.teikyo-u.ac.jp/~surgery2/hernia.

[8] Griffith CA, Nyhus LM, Condon RE (1989). The Marcy repair of indirect inguinal hernia: 1807 to present. Hernia.

[9] Gilbert AI, Graham MF, Voigt WJ (1999). A bilayer patch device for inguinal hernia repair. Hernia.

[10] Schumpelick V, Klinge U (2003). Prosthetic implants for hernia repair. Br J Surg.

[11] Vita GD, Milano S, Frazzetta M, Patti R, Palazzolo V, Barbera C (2000). Tension-free hernia repair is associated with an increase in inflammatory response against mesh. Am J Surg.

[12] Heise CP, Staring JR (1998). Mesh inguinodynia: a new clinical syndrome after inguinal herniorraphy?. J Am Coll Surg.

[13] Beets GL, Oosterhuis KJ, Go PM, Baeten CG, Kootsra G (1997). Longterm follow-up (12–15 years) of a randomized controlled trial comparing Bassini-Stetten, Shouldice, and high ligation with narrowing of the internal ring for primary inguinal hernia repair. J Am Coll Surg.

[14] Nilsson E, Haapaniemi S, Gruber G, Sandblom G (1998). Methods of repair and risk for reoperation in Swedish hernia surgery from 1992 to 1996. Br J Surg.

